# Islet environment and development of type 1 diabetes in the biobreeding rat model

**DOI:** 10.1038/s41392-025-02330-8

**Published:** 2025-08-05

**Authors:** Patricia Recio-López, Per-Olof Berggren, Montserrat Visa Majoral, Ismael Valladolid-Acebes, Lisa Juntti-Berggren

**Affiliations:** 1 The Rolf Luft Research Center for Diabetes and Endocrinology, Karolinska Institutet, Karolinska University Hospital, Stockholm, Sweden; 2 Center for Diabetes and Metabolism Research, Division of Endocrinology and Metabolism, West China Hospital, Sichuan University, Chengdu, China

**Keywords:** Physiology, Metabolic disorders

Dear Editor,

Type 1 diabetes (T1D) is a complex autoimmune disease characterized by the progressive destruction of pancreatic β-cells, leading to insulin deficiency. Despite extensive research, the specific triggering factors that initiate and advance T1D remain elusive. Over recent decades, we have observed a concerning global increase in T1D incidence, notable not only for its rising numbers, but also for a concerning trend towards younger age at diagnosis. Transplantation of pancreatic islets to the portal system of the liver is used as a treatment strategy for T1D. However, the success rate has been limited due to poor graft survival, despite immunosuppressive treatment, and often multiple transplantations are needed to obtain adequate secretion of insulin.^1^ Hence, for islet transplantation to become a viable treatment option for T1D, it is essential to both identify a more suitable transplantation site and develop methods to engineer islets that are resistant to inflammatory and immunological attacks.

In this study, we aimed to investigate the influence of the endogenous environment on the development of T1D using the BioBreeding (BB) rat model, as similar studies are not possible to do in humans. There are many similarities between BB rat and human T1D, but one key difference is that the BB rats are T cell lymphopenic due to a mutation in the *Gimap5* gene. The Diabetes Prone (DP) BB rats in our model are genetically predisposed and exhibit a 100% incidence of a human-like T1D with an average onset around 60 days of age. In contrast, their Diabetes Resistant (DR) counterparts do not develop diabetes.^2^ By using the technique of transplanting pancreatic islets to the anterior chamber of the eye,^3^ it was possible for the first time to study in vivo the effects of a prediabetic environment on healthy islets and vice versa, longitudinally, and non-invasively.

At 25 days of age, when the DP BB rats were still prediabetic and exhibiting normal glucose levels, we cross-transplanted islets from DP rats into the anterior chamber of the eye of healthy DR rats, and DR islets into DP rats (Fig.1a). Three weeks post-transplantation, we performed the initial imaging assessments of islet vascularization within the anterior chamber of the eye using laser scanning confocal microscopy. Our observations confirmed vascularization of all transplanted islets at this stage (Fig. 1a). However, two weeks later, as the DP BB rats approached the onset age for T1D, there was an almost complete loss of functional vessels in the transplanted DR islets (Fig. 1a).

**Fig. 1:**
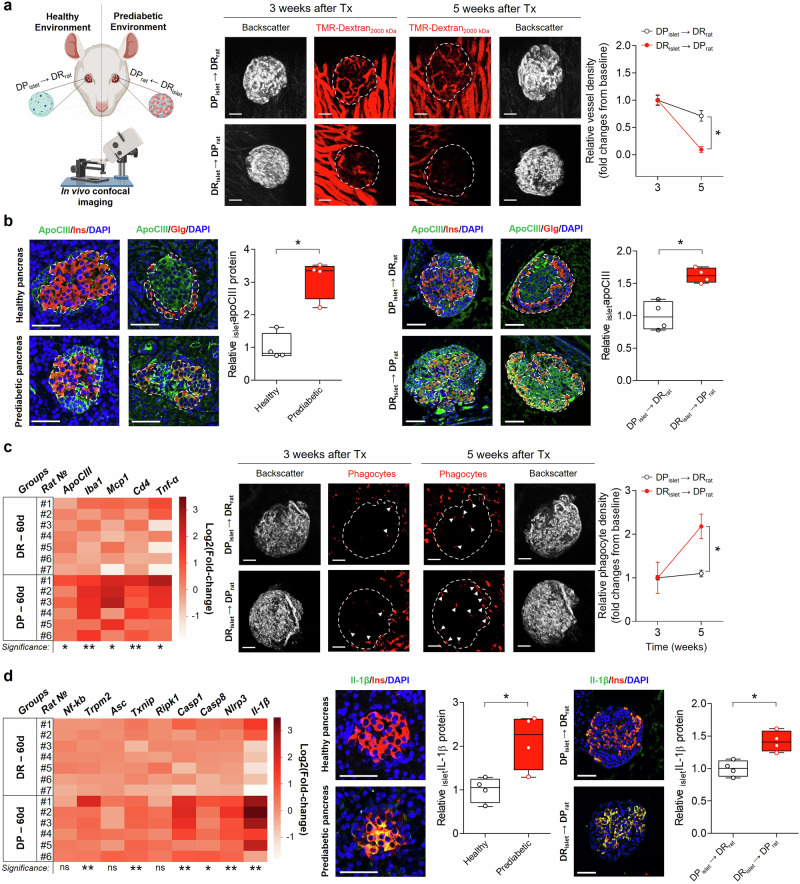
Effects of cross-transplanting healthy islets to a prediabetic environment and vice versa. **a** A schematic overview of the experimental protocol followed, to the right, by in vivo confocal representative images three and five weeks after transplantation of islets into the anterior chamber of the eye. Diabetes prone islets were transplanted to healthy recipients (DP_islet_ → DR_rat_, top) and healthy islets to prediabetic recipients (DR_islet_ → DP_rat_, bottom). Islet vasculature was visualized by intravenous injection of TMR-dextran_2000 kDa_ and vessel density, relative to baseline, was quantified volumetrically. Data are shown as mean ± SEM of *N* = 4 recipients per group. 2-ANOVA followed by Bonferroni´s posthoc test was used. *p < 0.05 for comparisons between experimental groups. **b** To the left, immunostaining of apoCIII, insulin, glucagon and DAPI in pancreatic sections from healthy (top) and prediabetic (bottom) rats, and to the right from transplanted islets in the the anterior chamber of the eye (DP_islet_ → DR_rat_, top; DR_islet_ → DP_rat_, bottom). In the boxplots, beside the images, a quantitative volumetric analysis of apoCIII protein in islets from the respective groups is presented, *N* = 4. **c** To the left, heatmap showing the expression levels of apoCIII and inflammatory markers by qRT-PCR in isolated islets from 60 days old DP and DR rats, *N* = 6–7. In the middle, intra-islet infiltration of phagocytes, indicated by white triangles, is shown 24 h after the injection of TMR-dextran_2000 kDa_ in DP_islet_ → DR_rat_ (top) and DR_islet_ → DP_rat_ (bottom).To the right, phagocyte density, relative to baseline, was quantified volumetrically. Data are shown as mean ± SEM of *N* = 4 recipients per group. 2-ANOVA followed by Bonferroni´s posthoc test was used, *p < 0.05 for comparisons between experimental groups. **d** To the left, heatmap showing the qRT-PCR expression levels of inflammasome-related genes in isolated islets from 60-day-old DP and DR rats. Overlay of insulin, IL-1β and DAPI immunostaining in islets in pancreatic sections, shown in the center, from healthy (top) and prediabetic (bottom) rats, and to the right in transplanted islets in the anterior chamber of the eye (DP_islet_ → DR_rat_, top; DR_islet_ → DP_rat_, bottom). In the boxplots, beside the images, quantitative volumetric analysis of IL-1β protein in islets from the respective groups are shown, *N* = 4. In (**c**, **d**) the vertical axis to the right of the heatmaps represents the log2 fold changes. In (**b**, **d**) the relative protein data are shown as a Tukey’s boxplot and a non-parametric two-tailed Mann-Whitney T-test was used, *p < 0.05. Scale bars are 50 µm in all microphotographs. The schematic overview was created using BioRender.com

Apolipoprotein CIII (apoCIII) is a small protein mainly produced in the liver, and known for its pro-inflammatory and diabetogenic properties.^4^ In normolipidemic individuals with T1D, increased circulating levels of apoCIII correlate with a higher risk of cardiovascular disease, and there is an independent relationship between elevated apoCIII levels and microvascular disease complications in patients with T1D.^4^ Furthermore, haplotypes in the apoCIII gene, leading to augmented levels of apoCIII, are associated with an increased susceptibility to T1D.^4^ In addition, we have shown that when β-cells were exposed to serum from newly diagnosed T1D patients, it induced Ca^2+^-mediated apoptosis, and the responsible factor was identified to be apoCIII.^5^

We have previously shown that apoCIII is present in mouse and human islets, and here we confirm its presence in rat islets as well (Fig. 1b). Immunostaining revealed that apoCIII is expressed in both α- and β-cells (Fig. 1b). In pancreatic sections, there were more apoCIII-positive cells in islets from prediabetic rats, close to onset of T1D, compared to islets from healthy rats (Fig. 1b). This was also true for islets in the anterior chamber of the eye, where a prediabetic environment increased the expression of apoCIII in originally healthy islets (Fig.1b). Furthermore, the percentage of β-cells were decreased in the prediabetic islets, both in the pancreas and in the transplanted islets in the anterior chamber of the eye (pancreas: DP, 53.7 ± 8.5% versus DR, 75.5 ± 3.4%, *p* < 0.05; eye: DP, 33.6 ± 4.2% versus DR, 56.8 ± 3.3%, *p* < 0.05).

We further analyzed the expression of several inflammatory genes in islets from 60-days-old rats. ApoCIII was upregulated in DP islets and this upregulation was accompanied by an increase in phagocytes and immune cell infiltration markers (*Iba1*, *Mcp1* and *Cd4*) and elevated *Tnf-α* as signs of ongoing inflammation (Fig. 1c). Most inflammasome-related genes were upregulated in prediabetic islets, thus resulting in a rise in *Il1-β* (Fig. 1d). Immunostaining of pancreatic sections corroborated these findings, revealing elevated levels of IL-1β in DP rat islets compared to those from DR rats (Fig. 1d). Similar patterns were observed in DR islets transplanted into the anterior chamber of the eye of DP animals, suggesting that islets in the eye serve as reliable indicators of pancreatic islet behavior (Fig. 1d).

In summary, our study utilizing the anterior chamber of the eye platform demonstrates that while transplanted islets can initially engraft and vascularize, it is predominantly the surrounding in vivo environment that dictates their fate, emphasizing the need for a strategic approach to islet transplantation in T1D patients.

Based on our extensive prior and ongoing studies, we propose the anterior chamber of the eye as a promising novel transplantation site in T1D. To improve the survival of transplanted islets, it is crucial to engineer them to resist inflammatory and immunological challenges. By taking the advantage of the anterior chamber of the eye as a transplantation site and the cornea as a natural body window for imaging, we could in the present letter clearly show that the environment has major impact on graft survival. Moreover, we could demonstrate that survival of the pancreatic islets was negatively correlated to the expression levels of apoCIII. Since our previous work has shown that reducing apoCIII levels in vivo with antisense oligonucleotides can delay T1D onset we propose this apolipoprotein to be a tentative target when trying to combat the inflammatory/immunological reactions associated with islet transplantation. Our next objective is to generate human islet organoids with reduced apoCIII using the CRISPR/Cas9 technique, and expose them to a diabetogenic environment to assess whether this enhances their resistance.

## Supplementary information

Supplemental material

## Data Availability

All data supporting the findings of this study are available in the main text and its supplementary information. Additional data related to this study may be requested from the authors.
