# Supplementary material for: Islet environment and development of type 1 diabetes in the biobreeding rat model
Source: Signal Transduct Target Ther. 2025 Aug 5;10:247. doi: 10.1038/s41392-025-02330-8 (PMC12322098; doi:10.1038/s41392-025-02330-8)
Supplement: Supplementary file 1 — Supplemental material [file 41392_2025_2330_MOESM1_ESM.docx]

Supplementary Materials for

**Islet environment and development of type 1 diabetes in the biobreeding rat model**

Patricia Recio-López^1^, Per-Olof Berggren^1,2^, Montserrat Visa Majoral^1^, Ismael Valladolid-Acebes^1^ and Lisa Juntti-Berggren^1^

Correspondence to: [ismael.valladolid.acebes@ki.se](mailto:ismael.valladolid.acebes@ki.se)

**This PDF file includes:**

Materials and Methods

Materials and Methods

**Animals**

BioBreeding (BB) rats were obtained from our breeding colony at Karolinska Institutet. The incidence of diabetes among our diabetes prone (DP) BB rats is 100% and the age of onset is on average 60 days. Diabetes onset is defined as blood glucose levels ≥ 15 mmol/L for three consecutive days. Diabetes resistant (DR) BB rats are healthy controls and do not develop diabetes.

The animals were housed under specific pathogen-free conditions in a temperature- and humidity-controlled room with 12 h light:dark cycles. They were fed R70 chow diet (Lantmännen, Sweden) and water *ad libitum*. All animal care and experiments were carried out according to the Animal Experiment Ethics Committee at Karolinska Institutet. Rats were 25 days old at start of the experiments, unless otherwise specified. Both male and female rats were included.

**Isolation of islets and transplantation into the anterior chamber of the eye**

Donor rats were sacrificed with CO_2_ and pancreas was perfused with 1mg/mL collagenase A (Roche) diluted in Hank’s balanced salt solution (HBSS; Thermo Fisher Scientific) supplemented with 0.5 % (w/v) bovine serum albumin (BSA; Sigma-Aldrich), 25 mM HEPES (Thermo Fisher Scientific) and buffered at pH 7.4 (HBSS-BSA buffer). After pancreas distention, a total pancreatectomy was performed and the pancreas was digested at 37°C in a bath with continuous gentle shaking for 13 min. Digestion was stopped by adding ice-cold HBSS-BSA buffer. Islets were isolated from the exocrine fraction by handpicking under a stereoscopic microscope. Isolated islets were cultured in RPMI 1640 medium (Thermo Fisher Scientific) supplemented with 10 % (v/v) fetal bovine serum (FBS; Gibco), 2 mM l-glutamine (Gibco) and penicillin-streptomycin (100 U/ml of penicillin and 100 pg/ml of streptomycin; Gibco). Islets were kept at 37°C under a 5 % CO_2_ and 95 % air-humidified atmosphere for a recovery time of 24 hours prior to transplantation. Recipient rats were anesthetized with isoflurane (2.5 - 3 %) diluted in oxygen (400 - 500 mL/min). Isolated islets were transplanted into the anterior chamber of the eye as previously described.[3]

***In vivo* imaging of transplanted islets and image analysis**

Transplanted islets were imaged *in vivo* for vascularization and infiltration of phagocytes using a TCS SP5 II laser scanning confocal microscope (Leica Microsystems) with water-dipping objectives (Leica HCX IRAPO L 25.0 x 0.95 and HXC-APO10x/0.30 NA). Viscotears (Bausch+Lomb) was used as the immersion liquid between the objective and the eye. Backscatter reflected light images were obtained using a 633 nm laser beam. For visualization of islet vascularization, animals were anesthetized with isoflurane (Baxter, USA). 150 μl of PBS solution containing 3.3 mg/ml of 2000 kDa tetramethylrhodamine- (TMR) conjugated dextran (Thermo Fisher Scientific, USA) was injected intravenously prior to imaging. Z-stacks of 3 μm thickness were acquired for every islet graft (Ex.: 561 and 594 nm, Em.: 558–564 nm). Blood vessels were imaged directly after injection, whereas phagocytes were imaged 24 h later. Unprocessed original images were used in all quantifications of *in vivo* vascularization and phagocyte infiltration. Graft volume was estimated from backscatter signals using Volocity image analysis software (Perkin Elmer, USA). Dextran-labeled islet capillary and phagocyte density were identified using the same thresholds for all groups at all time points, and structures smaller than 10 μm^3^ were automatically excluded. Vessel and phagocyte density of each islet graft at 3- and 5-weeks post-transplantation was calculated from dividing the volume of islet vessels or phagocytes by the estimated graft volume and presented as fold-changes from baseline (3 weeks after transplantation) in each group.

**RNA isolation, cDNA preparation and quantitative real-time PCR (qRT-PCR)**

Total RNA was isolated from lysates of isolated pancreatic islets using the RNeasy Lipid Tissue Mini Kit (Qiagen), according to the manufacture’s protocol. Total RNA concentrations were determined using a NanoPhotometer P330 (IMPLEN). 500 ng of total RNA from islets was used for cDNA preparation using the High-Capacity cDNA Reverse Transcription Kit (Applied Biosystems), following manufacturer’s instructions.

qRT-PCR was performed in a QuantStudio5 PCR system thermal cycler (Applied biosystems) with Power-Up SYBR green PCR master mix (Applied biosystems). Analysis of gene expression was done with the ΔΔCt method. The log2 fold change in gene expression was calculated relative to the DR group. The NumPy, Matplotlib, and Seaborn libraries were used to generate a heatmap and analyze the differential expression of target genes between DP and DR rats. A scale bar adjacent to the heatmap represents log2 fold change values, with color gradients indicating upregulation (red) and downregulation (white). As control gene we used TATA-binding protein (TBP). Primer sequences are available upon request.

**Immunostaining**

Tissues for immunostaining were dissected out and fixed overnight in 4 % (v/v) paraformaldehyde (Sigma-Aldrich) at room temperature and at 60 × *g* rotation speed. After fixation, they were dehydrated with ethanol gradient, cleared with xylol, and paraffin-embedded. Five µm thick sections underwent a standard fluorescent immunohistochemistry method for paraffin sections. Images were acquired using Leica TCS SP8 confocal microscope (Leica Microsystems) using a 20× glycerol immersion objective. For each image, optical sections were taken every 0.5 µm. Nuclei were stained with 4´,6-diamidimo-2-phanylindole (DAPI; Mobitec) and slides containing the stained sections were mounted using a permanent mounting medium (Vector Laboratories).

Positively stained cells and volume of voxels were quantified using Volocity Software (x64; Improvision).

**Statistical analyses**

Statistical analyses were performed using GraphPad Prism 5.0. Data are presented as mean ± SEM. Statistical comparisons between two groups were performed using the non-parametric two-tailed Mann-Whitney U t-test. Two-ways ANOVA followed by Bonferroni´s post-hoc test was used when doing multiple comparisons between time points in different groups. Statistical significance was defined as *p* < 0.05.
